# *S100P* as a potential biomarker for immunosuppressive microenvironment in pancreatic cancer: a bioinformatics analysis and in vitro study

**DOI:** 10.1186/s12885-023-11490-1

**Published:** 2023-10-18

**Authors:** Weiwei Hao, Yanyan Zhang, Jingwen Dou, Pu Cui, Jicun Zhu

**Affiliations:** 1 Department of gastroenterology, the First Affiliated Hospital of Zhengzhou University, Zhengzhou, 450052 Henan China; 2 Department of Pharmacy, the First Affiliated Hospital of Zhengzhou University, Zhengzhou, 450052 Henan China

**Keywords:** Pancreatic cancer, *S100P*, Tumor microenvironment, CD8 + T cell, Immunotherapy, Immunosuppressive

## Abstract

**Background:**

Immunosuppression is a significant factor contributing to the poor prognosis of cancer. *S100P*, a member of the S100 protein family, has been implicated in various cancers. However, its role in the tumor microenvironment (TME) of pancreatic cancer remains unclear. This study aimed to investigate the potential impact of *S100P* on TME characteristics in patients with pancreatic cancer.

**Methods:**

Multiple data (including microarray, RNA-Seq, and scRNA-Seq) were obtained from public databases. The expression pattern of *S100P* was comprehensively evaluated in RNA-Seq data and validated in four different microarray datasets. Prognostic value was assessed through Kaplan-Meier plotter and Cox regression analyses. Immune infiltration levels were determined using the ESTIMATE and ssGSEA algorithms and validated at the single-cell level. *Spearman* correlation test was used to examine the correlation between *S100P* expression and immune checkpoint genes, and tumor mutation burden (TMB). DNA methylation analysis was performed to investigate the change in mRNA expression. Reverse transcription PCR (RT-PCR) and immunohistochemical (IHC) were utilized to validate the expression using five cell lines and 60 pancreatic cancer tissues.

**Results:**

This study found that *S100P* was differentially expressed in pancreatic cancer and was associated with poor prognosis (*P* < 0.05). Notably, *S100P* exhibited a significant negative-correlation with immune cell infiltration, particularly CD8 + T cells. Furthermore, a close association between *S100P* and immunotherapy was observed, as it strongly correlated with TMB and the expression levels of *TIGIT*, *HAVCR2*, *CTLA4*, and *BTLA* (*P* < 0.05). Intriguingly, higher *S100P* expression demonstrated a negative correlation with methylation levels (cg14323984, cg27027375, cg14900031, cg14140379, cg25083732, cg07210669, cg26233331, and cg22266967), which were associated with CD8 + T cells. In vitro RT-PCR validated upregulated *S100P* expression across all five pancreatic cancer cell lines, and IHC confirmed high S100P levels in pancreatic cancer tissues (*P* < 0.05).

**Conclusion:**

These findings suggest that *S100P* could serve as a promising biomarker for immunosuppressive microenvironment, which may provide a novel therapeutic way for pancreatic cancer.

**Supplementary Information:**

The online version contains supplementary material available at 10.1186/s12885-023-11490-1.

## Introduction

Pancreatic cancer is a highly lethal disease worldwide with a mortality rate that closely parallels its incidence [1]. In the United States and Europe, it ranks as the third and fourth leading causes of cancer-related mortality, respectively. [2, 3]. Despite some improvement, the five-year relative survival rate remains low at only approximately 10% [4]. Early diagnosis and treatment have been shown to reduce surgical complications, disease recurrence, and clinical deterioration, and improve survival rates. Therefore, it is of paramount importance to determine the etiology mechanisms and explore more effective treatment strategies.

In recent years, immunotherapy and targeted therapies have brought revolutionary progress in the therapy of various cancer types, including melanoma [5], lung cancer [6], and colorectal cancer [7]. However, the results have not been promising in patients with pancreatic cancer. This difference may be correlated with the higher TME heterogeneity of pancreatic cancer, particularly its highly desmoplastic stroma and immunosuppressive cell populations [8, 9]. The TME has been shown to inactivate the ability of cytotoxic T cells to eliminate tumor cells [10], and CD8 + T cells, the central subpopulation of cytotoxic T lymphocytes, are primarily responsible for eliminating tumor cells [10]. Therefore, a comprehensive analysis of TME might reveal the mechanisms of resistance to immunotherapy, which may provide the basis for pancreatic cancer immunology and opportunities to improve survival [11]. Immunomodulatory factors are vital to immunotherapy, and more profound research on the TME of pancreatic cancer will provide a new theoretical basis for the development of immunotherapy.

Numerous studies have demonstrated that *S100P* is an oncogenic gene involved in many types of tumors, such as breast cancer [12], gastric cancer [13], and pancreatic cancer [14]. However, the current literature provides limited insights into the possible role of *S100P* in the regulation of the TME in pancreatic cancer. Therefore, this study was designed to explore the potential biological mechanisms and regulation of *S100P* in the pancreatic cancer microenvironment. The main objective of this study is to enhance our understanding of the immunomodulatory effects of *S100P* in pancreatic cancer, and potentially leverage its role in improving the effectiveness of immunotherapy.

## Materials and methods

### Data collection and pre-processing

In this study, multiple datasets were utilized to perform comprehensive analyses. Specifically, microarray, RNA-Seq, and scRNA-Seq datasets were selected to evaluate different aspects of the research question. The datasets were obtained from various public repositories, including the GEO (https://www.ncbi.nlm.nih.gov/gds/), the ArrayExpress (https://www.ebi.ac.uk/arrayexpress/), CCLE (https://sites.broadinstitute.org/ccle), and UCSC Xena (https://xenabrowser.net/datapages/). More specifically, GSE28735, GSE15471, GSE16515, GSE71729, and GSE155698 were retrieved from the GEO database, while E-MTAB-6134 was downloaded from ArrayExpress. RNA-Seq data were collected from cellular and tissue levels through CCLE and UCSC Xena, respectively. In line with a prior publication, the same procedures for microarray pre-processing were implemented [15].

### Overview of *S100P* in pan-cancer and pancreatic cancer

The expression of *S100P* was initially examined in 33 solid tumor types using the GEPIA tool (http://gepia.cancer-pku.cn/) [16]. To assess *S100P* expression at the cellular level, we analyzed its expression in 31 cancer types using CCLE data. We then validated the expression levels of *S100P* in four different microarray datasets (GSE28735, GSE15471, GSE16515, and GSE71729) using the “limma” package. Clinical characteristics were subsequently analyzed in relation to *S100P* expression levels, and survival statistics for *S100P* were calculated using the “survival” package in multiple cancer cohorts and further validated in E-MTAB-6134. To explore the role of *S100P* in pancreatic cancer progression, we examined its expression levels in different TNM stages and *KRAS* mutation states.

### Assessment of the correlation between *S100P* and tumor microenvironment

Firstly, the ESTIMATE algorithm [17] was employed to calculate the TumorPurity (proportion of cancer cells in the tumor tissue), StromalScore (proportion of stromal ingredient), ImmuneScore (proportion of immune ingredient), and ESTIMATEScore (sum of the ImmuneScore and StromalScore) for each pancreatic cancer sample. The correlation between these scores and *S100P* expression was subsequently evaluated. Next, single-sample gene set enrichment analysis (ssGSEA) was implemented to quantify the scores of 29 immune-related signatures in the datasets E-MTAB-6134 and GSE71729 via the “GSVA” package. The infiltration levels between the high- and low-expression groups were investigated based on the median of *S100P* expression. The correlation between *S100P* expression and CD8 + T cells was validated using five algorithms, including CIBERSORT, TIMER, MCP-counter, quanTIseq, and xCell. Additionally, the “ImmuneSubtypeClassifier” package was employed to calculate the six immune subtypes (C1: wound healing, C2: IFN-γ dominant, C3: inflammatory, C4: lymphocyte depleted, C5: immunologically quiet, and C6: TGF-β dominant) [18]. The correlation between these immune subtypes and *S100P* expression was evaluated using previously processed data [15].

### Analysis of *S100P* in pancreatic cancer using scRNA-Seq data

*S100P* expression in the TME of pancreatic cancer was validated at the single-cell level using CRA001160 and GSE154778, with analysis conducted through the TISCH2 web (http://tisch.comp-genomics.org/) [19]. Then, we downloaded and reanalyzed the GSE155698 dataset, which includes seventeen primary pancreatic cancer samples. The dataset was subjected to quality control and processed using standardized protocols in the “Seurat” and “DoubletFinder” packages. The “harmony” package was also utilized to correct batch effect; the identities of cell types were characterized based on CellMarker 2.0 webserver (http://bio-bigdata.hrbmu.edu.cn/CellMarker/) [20]. Finally, to validate the relationship between the immune cell infiltration and *S100P* expression at the single-cell level, the proportions of these cells were calculated according to the *S100P* expression level.

### Examining the relationship between *S100P* expression and immunotherapy

Previous results have indicated an inverse correlation between *S100P* and CD8 + T cells, highlighting the potential of *S100P* in immunotherapy. Hence, we used two datasets, E-MTAB-6134 and TCGA-PAAD, to explore the correlation between *S100P* expression and immune checkpoint molecules (PD-1*/CD274*, *CTLA4*, *IDO1*, *BTLA*, *LAG3*, TIM-3*/HAVCR2*, *TIGIT*). The RNA-Seq data from TCGA was also utilized to compute the tumor mutation burden (TMB), and the *Spearman* correlation coefficient was used to determine the correlation between *S100P* expression and TMB expression. Finally, patients with pancreatic cancer were divided into high and low *S100P* expression groups according to the median value. We utilized “maftools” packages to observe the difference in mutated genes between the two groups [21].

### Exploring the correlation between *S100P* expression and DNA methylation levels

Investigating the potential causes of the increased *S100P* expression in patients with pancreatic cancer is of paramount importance. DNA methylation is acknowledged as a well-established mechanism for regulating gene expression [22]. Accordingly, we sought to investigate the relationship between DNA methylation and the expression of *S100P* by utilizing the MEXPRESS database (https://www.mexpress.be/). Furthermore, we aimed to explore the association between significant DNA methylation and infiltration levels of CD8 + T cells using *Spearman* correlation algorithm.

### Cell culture, RNA extraction, and reverse transcription PCR (RT-PCR)

This study extracted total RNA from five types of pancreatic cancer cell lines (PANC-1, SW1990, BxPC-3, Capan 1, and AsPC-1), and its quality was assessed using an ND-1000 spectrophotometer (NanoDrop Technologies; Thermo Fisher Scientific, Inc). Next, cDNA was synthesized, and RT-PCR was performed using Applied Biosystems (Thermo Fisher Scientific, Inc). The primer sequences used were as follows: *S100P*, forward, 5’‑GCACCATGACGGAACTAGAGACA‑3’ and reverse, 5’‑CAGGTCCTTGAGCAATTTATCCAC‑3’; *GAPDH*, forward, 5’‑GCACCGTCAAGGCTGAGAAC‑3’ and reverse, 5’‑TGGTGAAGACGCCAGTGGA‑3’. Finally, the relative expression abundance was determined using the ΔCt(*S100P*-*GAPDH*) method.

### Immunohistochemistry

Thirty patients with a pathological diagnosis of pancreatic cancer underwent radical surgery, and thirty adjacent tissues were carried out to assess the S100P expression at the protein level. First, the sections were deparaffinized and rehydrated. Next, it was incubated with polyclonal anti-S100P antibody (1:10000 dilution) (proteintech, 11283-1-AP), log2(H-score) was used to quantify S100P expression from immunohistochemistry images. Then, the expression of S100P protein was assessed in 137 primary tumors and 74 normal tissues using the UALCAN platform (http://ualcan.path.uab.edu/index.html) [23].

## Results

### Pan-cancer expression pattern, prognostic significance, and clinical correlation of *S100P*

**Fig. 1:**
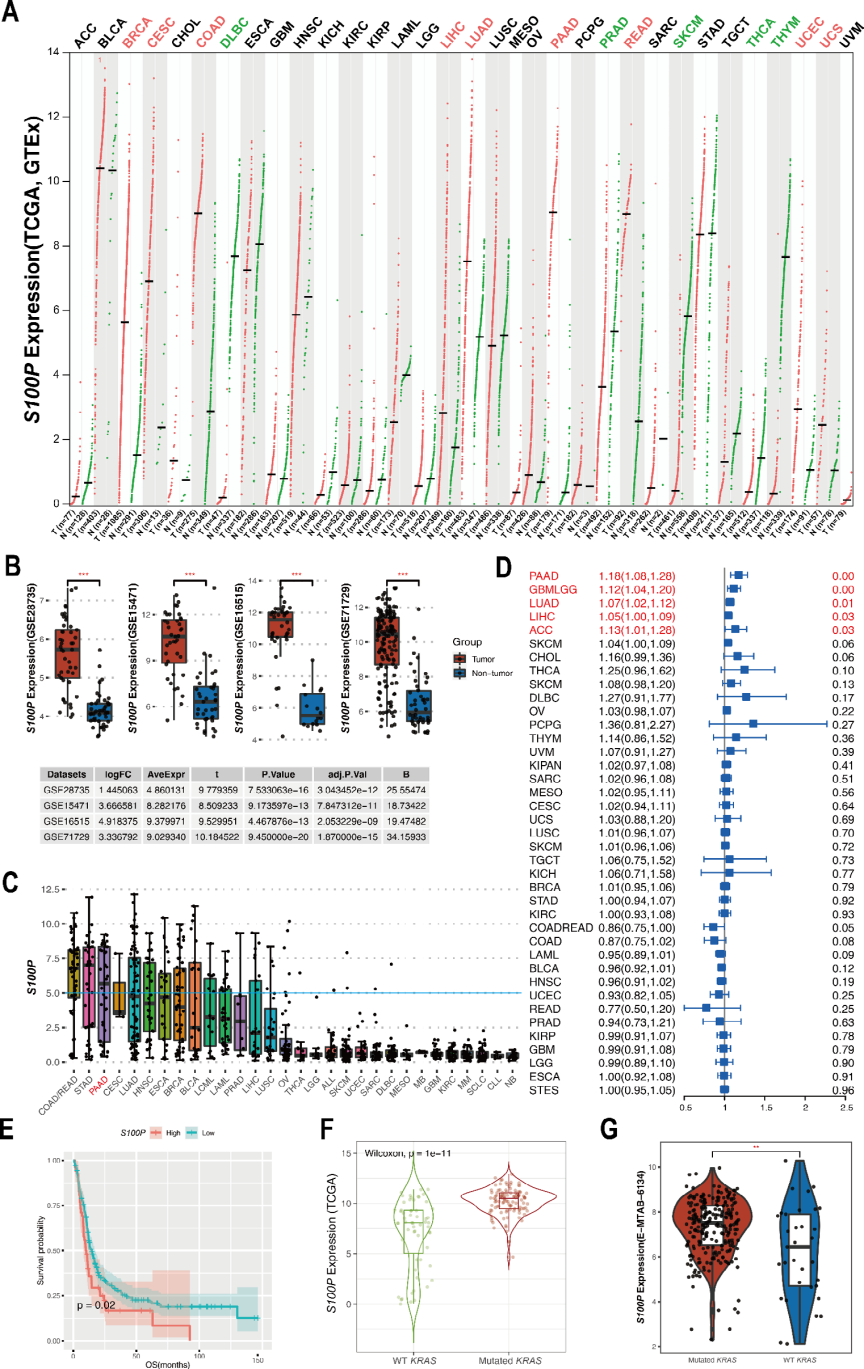
Overview of *S100P* in human cancers. (**A**) Assessment of *S100P* expression in tumor and adjacent tissues using the TCGA and GTEx databases. (**B**) Validation of *S100P* expression in GSE28735, GSE15471, GSE16515, and GSE71729. (**C**) Evaluation of *S100P* expression in pan-cancer cells using the CCLE dataset. (**D**) Estimation of the prognostic value of *S100P* in different cancer types using cox regression analysis. (**E**) Validation of the prognostic value of *S100P* using the E-MTAB-6134 dataset. (**F-G**) Comparison of *S1000P* expression in pancreatic cancer samples with different *KRAS* mutation statuses using the TCGA-PAAD and E-MTAB-6134 databases. ^**^: *P* < 0.01, ^***^: *P* < 0.001

In pan-cancer, it was found that *S100P* was more highly expressed in breast cancer(BRCA), cervical cancer(CESC), colon cancer(COAD), liver cancer(LIHC), lung adenocarcinoma(LUAD), pancreatic cancer(PAAD), rectal cancer(READ), endometrioid cancer(UCEC), and uterine carcinosarcoma(UCS), but lower in large B-cell lymphoma(DLBC), prostate cancer(PRAD), melanoma(SKCM), thyroid cancer(THCA), and thymoma(THYM) (Fig. 1A), especially in PAAD (Figure S1). Four microarray datasets provided validation results that supported the notion that tumor tissues exhibited higher expressions of *S100P* as compared to non-tumor samples (Fig. 1B). Moreover, cellular analysis of *S100P* expression among thirty different types of cancer cells showed a significant trend of high expression in PAAD (Fig. 1C). Survival analysis identified that higher expression of *S100P* was statistically linked with poor survival in five cancers (Fig. 1D), including PAAD, glioma(GBMLGG), LUAD, LIHC, adrenocortical carcinoma(ACC) with 1.18(95% CI 1.08,1.28), 1.12(95% CI 1.04,1.20), 1.07(95% CI 1.02,1.12), 1.05(95% CI 1.00,1.09), 1.13(95% CI 1.01,1.28), significantly in PAAD, and this finding was validated in E-MTAB-6134 with identical results (Fig. 1E). In the clinical aspect, the expression of *S100P* was associated with *KRAS* mutation in TCGA-PAAD and E-MTAB-6134 datasets (Fig. 1F&G). Notably, the mRNA expression of *S100P* was significantly increased in patients with stage II-IV compared to patients with stage I, suggesting that *S100P* may potentially contribute to the progression of pancreatic cancer (Figure S2). Furthermore, according to the pathway analysis from TISIDB, *S100P* was involved in three pathways: immune system, innate immune system, and neutrophil degranulation (http://cis.hku.hk/TISIDB/browse.php?gene=S100P).

### *S100P* expression in pancreatic cancer negatively correlates with immune infiltration

**Fig. 2:**
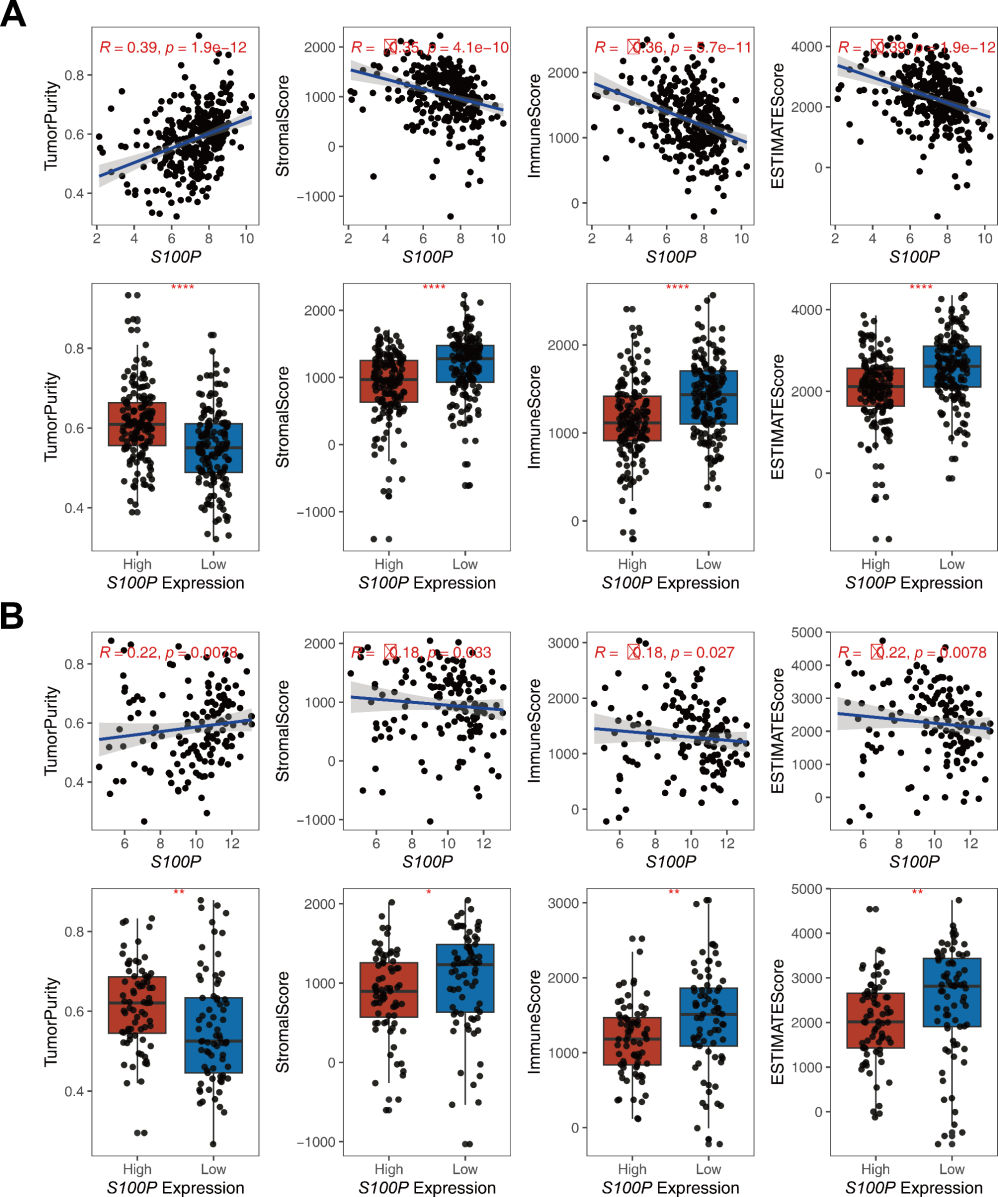
The correlation between *S100P* expression and Tumor Purity, Stromal Score, Immune Score, and ESTIMATE Score, as well as differential analysis based on the median of *S100P* expression. (**A**) E-MTAB-6134 dataset. (**B**) GSE71729 dataset. TumorPurity: proportion of cancer cells in the tumor tissue, StromalScore: proportion of stromal ingredient, ImmuneScore: proportion of immune ingredient, ESTIMATEScore: sum of the ImmuneScore and StromalScore. ^*^: *P* < 0.05, ^**^: *P* < 0.01, ^***^: *P* < 0.001

**Fig. 3:**
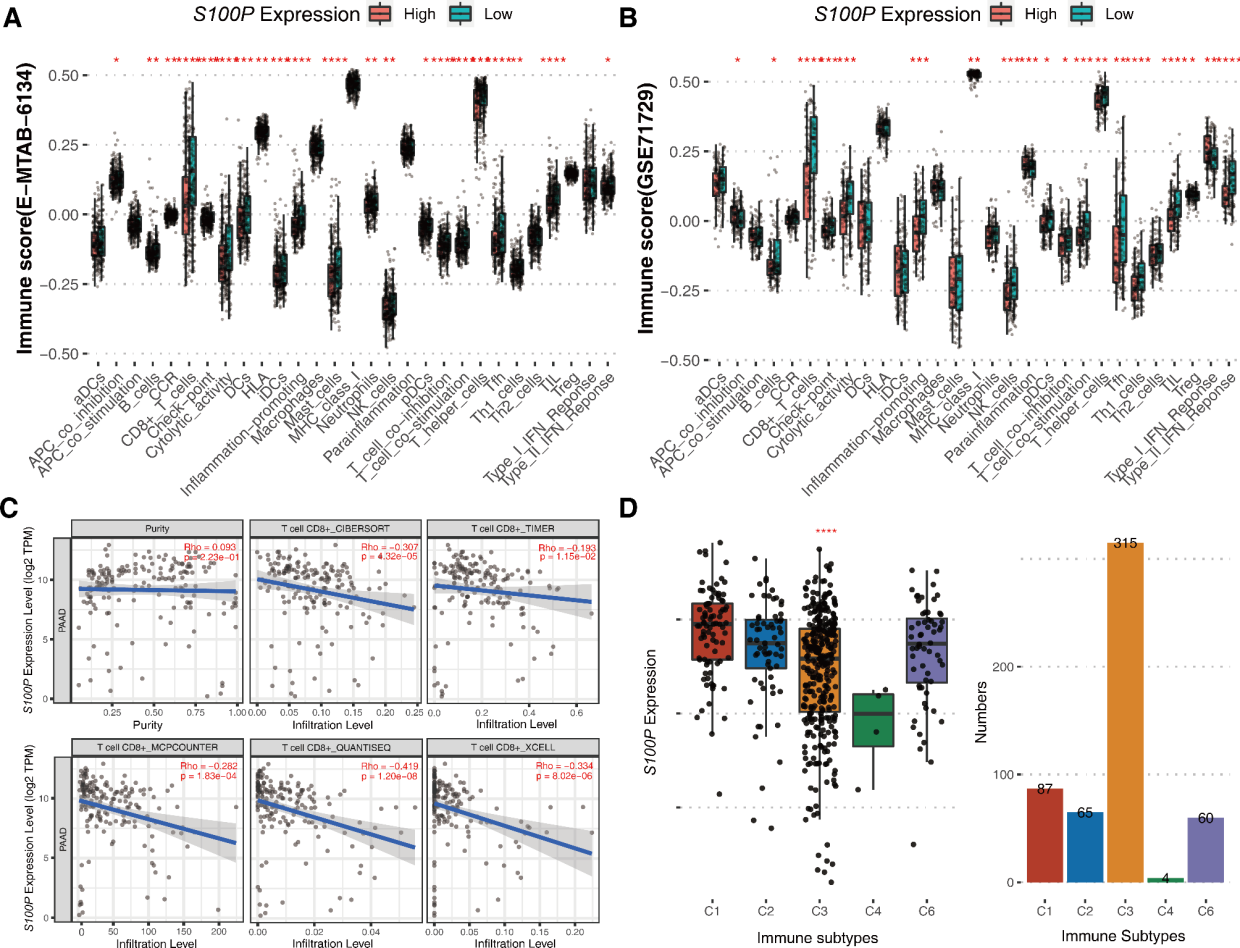
The landscape of the TME in pancreatic cancer and the characteristics of different *S100P* subgroups. (**A-B**) Proportions of various TME cells were analyzed using E-MTAB-6134 and GSE71729 datasets, and the immune score of the two subgroups is illustrated by scattered dots. (**C**) The correlation between *S100P* expression level and CD8 + T cells in pancreatic cancer using five different algorithms. (**D**) The analysis of immune subtypes was conducted using E-MTAB-6134 dataset. ^*^: *P* < 0.05, ^**^: *P* < 0.01, ^***^: *P* < 0.001

After calculating TumorPurity, StromalScore, ImmuneScore, and ESTIMATEScore, a subsequent analysis revealed a negative correlation between *S100P* and StromalScore, ImmuneScore, and ESTIMATEScore, whereas a positive correlation was observed with TumorPurity in E-MTAB-6134 dataset (Fig. 2A). This pattern was similarly observed in GSE71729 dataset (Fig. 2B). Notably, immune scores indicated that samples with high levels of *S100P* expression had significantly lower immune-infiltrating values. This trend was particularly notable in CD8 + T cells in E-MTAB-6134 and GSE71729 datasets (Fig. 3A**&B**). Five methods were used to validate the above findings, all of which revealed a significant negative association between *S100P* expression in pancreatic cancer tissues and CD8 + T cell infiltration. The correlation coefficients obtained were − 0.307, -0.193, -0.282, -0.419, and − 0.334, respectively (Fig. 3C). Furthermore, patients with pancreatic cancer were predominantly enriched in the C3 subtype, in which low expression of *S100P* was a noteworthy trend (Fig. 3D). Finally, we employed RNA-Seq data from TCGA to verify these findings and found consistent results through TISIDB (Figure S3).

### *S100P* expression in pancreatic cancer associated with tumor microenvironment at the single cell level

**Fig. 4:**
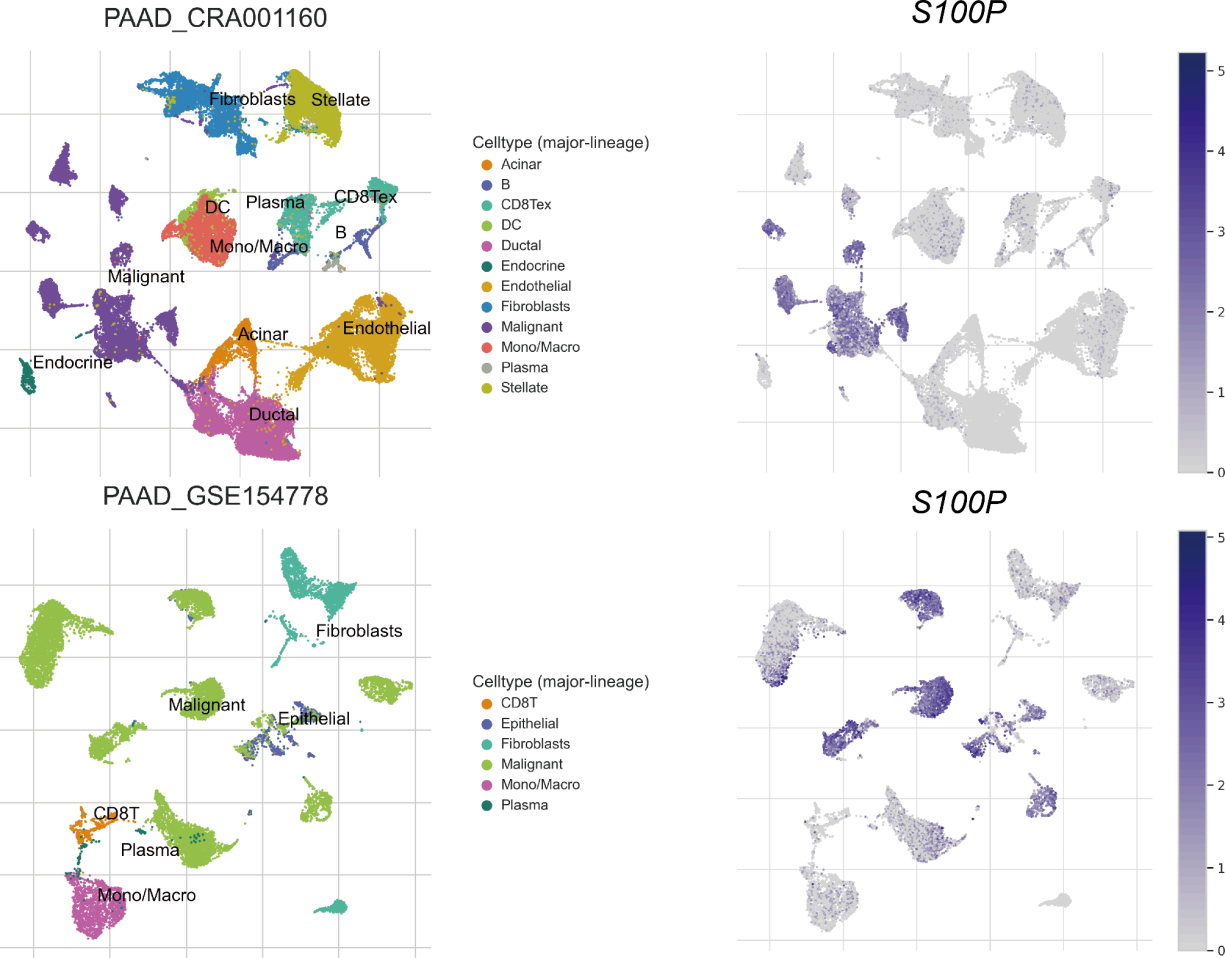
Analysis of *S100P* expression and immune cell infiltration in CRA111160 and GSE154778. (left) The UMAP plots of diverse cell types in pancreatic cancer tissues colored by major cell lineage. (right) Expression distribution of *S100P* in all cell types

**Fig. 5:**
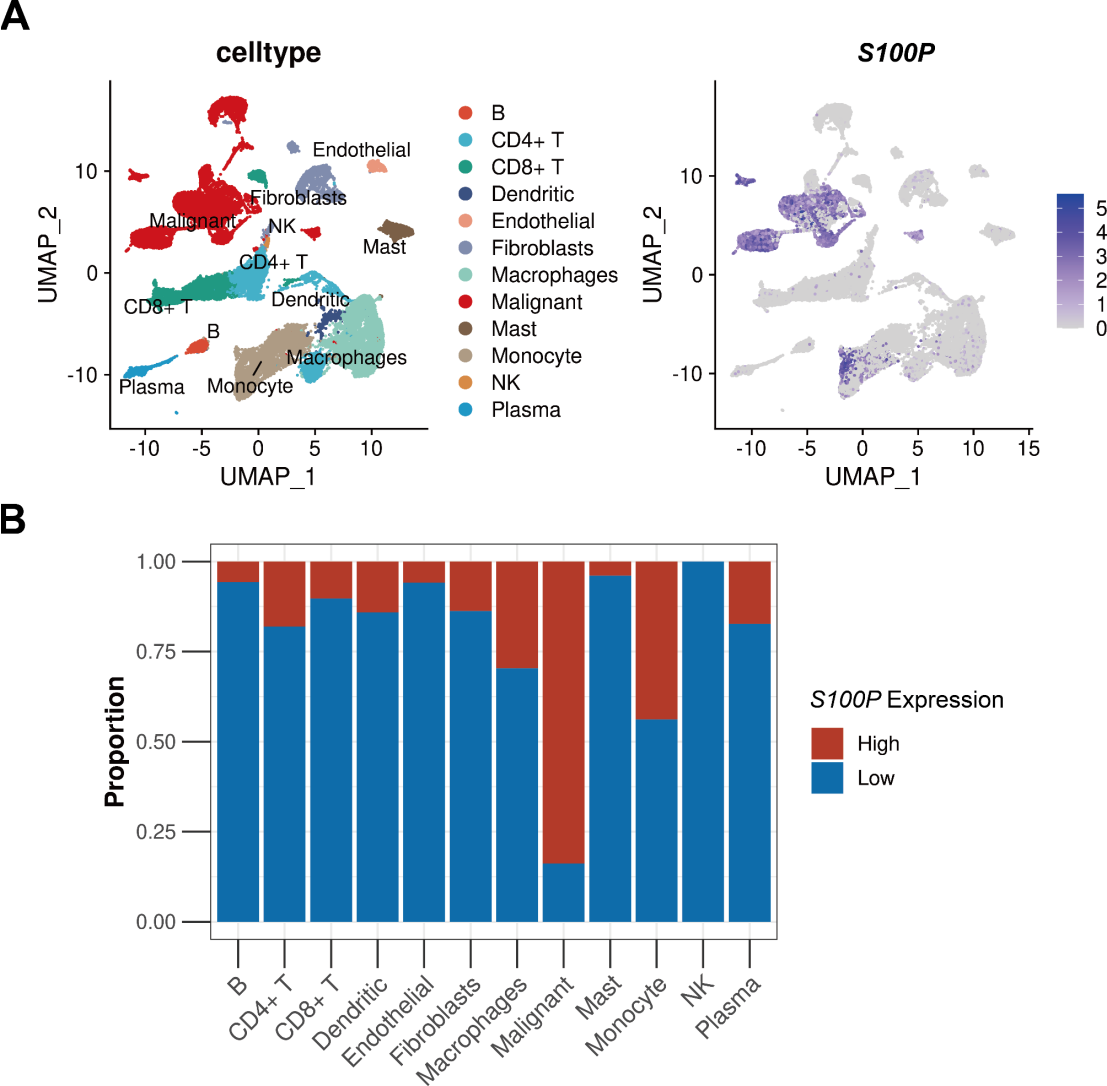
Analysis of *S100P* expression and immune cell infiltration in GSE155698. (**A**) The UMAP plot of diverse cell types in pancreatic cancer(left) and the expression of *S100P* in all cell types(right). (**B**) The proportion of all cell types in high and low *S100P* groups

At the single-cell level, *S100P* has been found to be closely associated with the TME of pancreatic cancer, as demonstrated by the results of CRA001160 and GSE154778 datasets. The expression of *S100P* was significantly higher in malignant cells compared to non-malignant cells in these datasets (Fig. 4). Further analysis of the GSE155698 dataset, which included 41,378 cells from 17 tumor samples, revealed that high expression of *S100P* was also predominantly observed in malignant cells (Fig. 5A). Interestingly, the percentage of immune-related cells, such as B cells, CD4 + T cells, CD8 + T cells, dendritic cells, endothelial cells, macrophages, mast cells, monocytes, NK cells, and plasma cells, was significantly lower in the group with higher expression of *S100P* compared to the group with low expression. Conversely, the proportion of malignant cells was higher in the group with high *S100P* expression (Fig. 5B).

### Higher *S100P* expression correlates with reduced response to immunotherapy

**Fig. 6:**
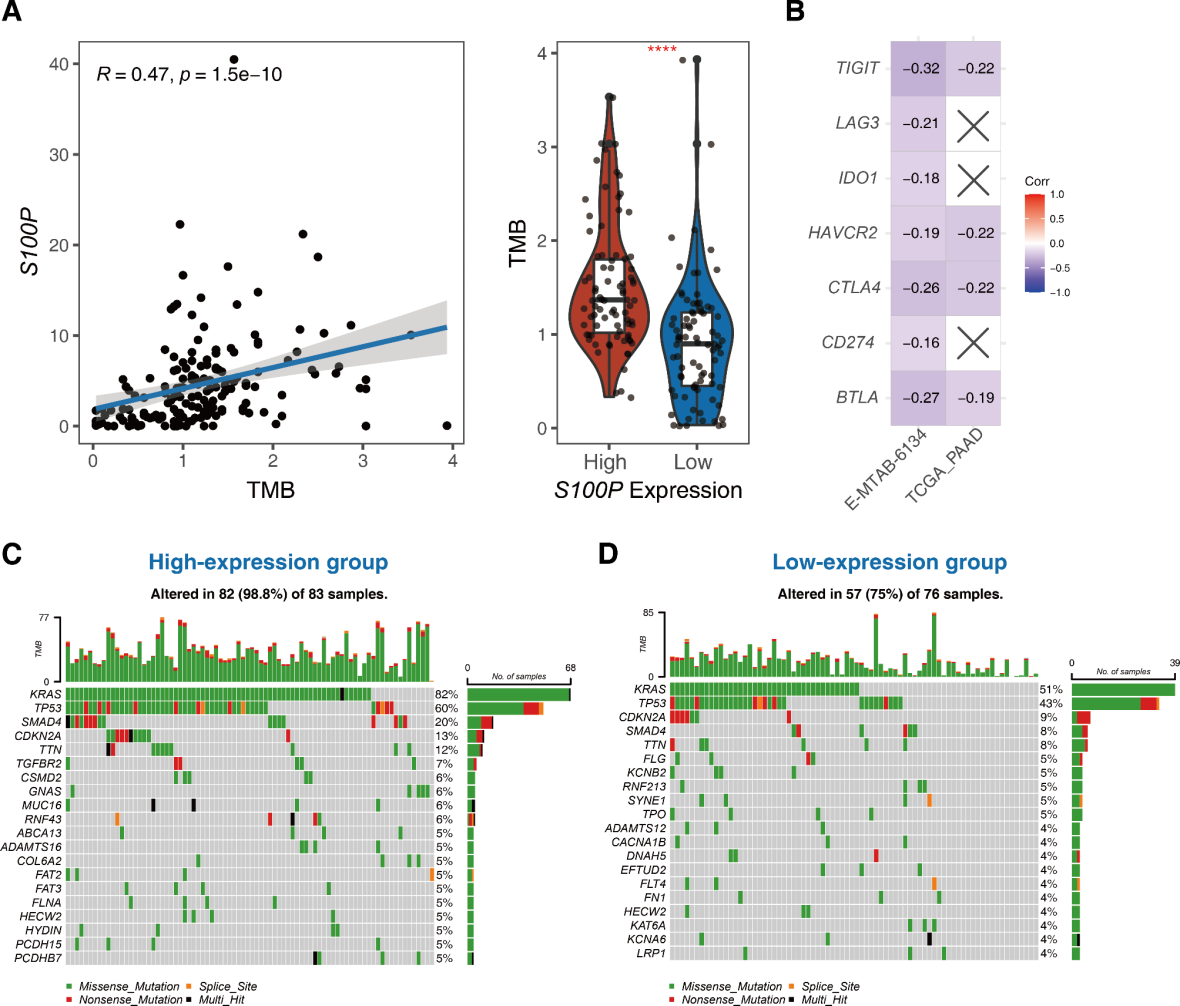
The association between *S100P* and immunotherapy. (**A**) The correction between *S100P* and TMB in pancreatic cancer. (**B**) The heatmap of *S100P* and immune checkpoint molecules expression level in E-MTAB-6134 and TCGA-PAAD datasets. (**C**) Waterfall plot of the top 20 most frequently mutated genes in *S100P* high- and low-expression groups. ^***^: *P* < 0.001

The results of the TMB analysis revealed a significant positive correlation between TMB and *S100P* expression (*R* = 0.47, *P* < 0.05); notably, the high *S100P* expression group had a higher TMB value compared to the low expression group (Fig. 6A). Moreover, the mutation genes for the two groups were found to be distinct (Fig. 6C**&D**). Analysis of the expression of immunotherapy-related genes (IRGs) revealed a negative correlation between *S100P* expression and *TIGIT*, *LAG3*, *IDO1*, *CTLA4*, PD-1/*CD274*, and *BTLA* in the E-MTAB-6134 dataset. In the TCGA-PAAD dataset, *S100P* expression was negatively correlated with *TIGIT*, *BTLA*, TIM-3*/HAVCR2*, *CTLA4*, and *BTLA* (Fig. 6B). These findings suggest that patients with higher *S100P* expression may not benefit from immunotherapy due to the down-regulation of immune checkpoints and reduced CD8 + T cell infiltration in this group.

### Overexpression of *S100P* negatively correlates with DNA methylation level

**Fig. 7:**
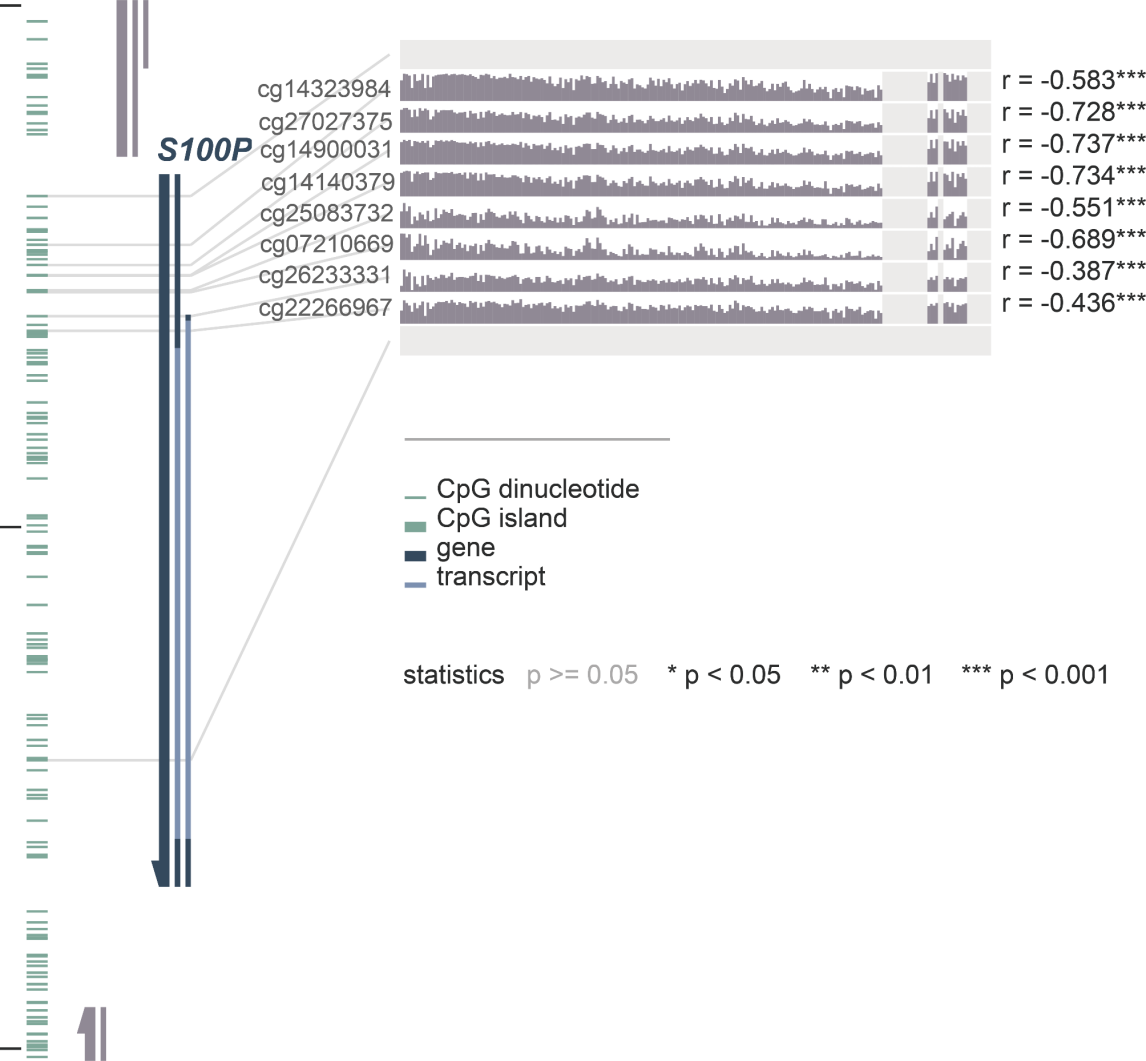
The correlation of *S100P* expression and DNA methylation level

**Fig. 8:**
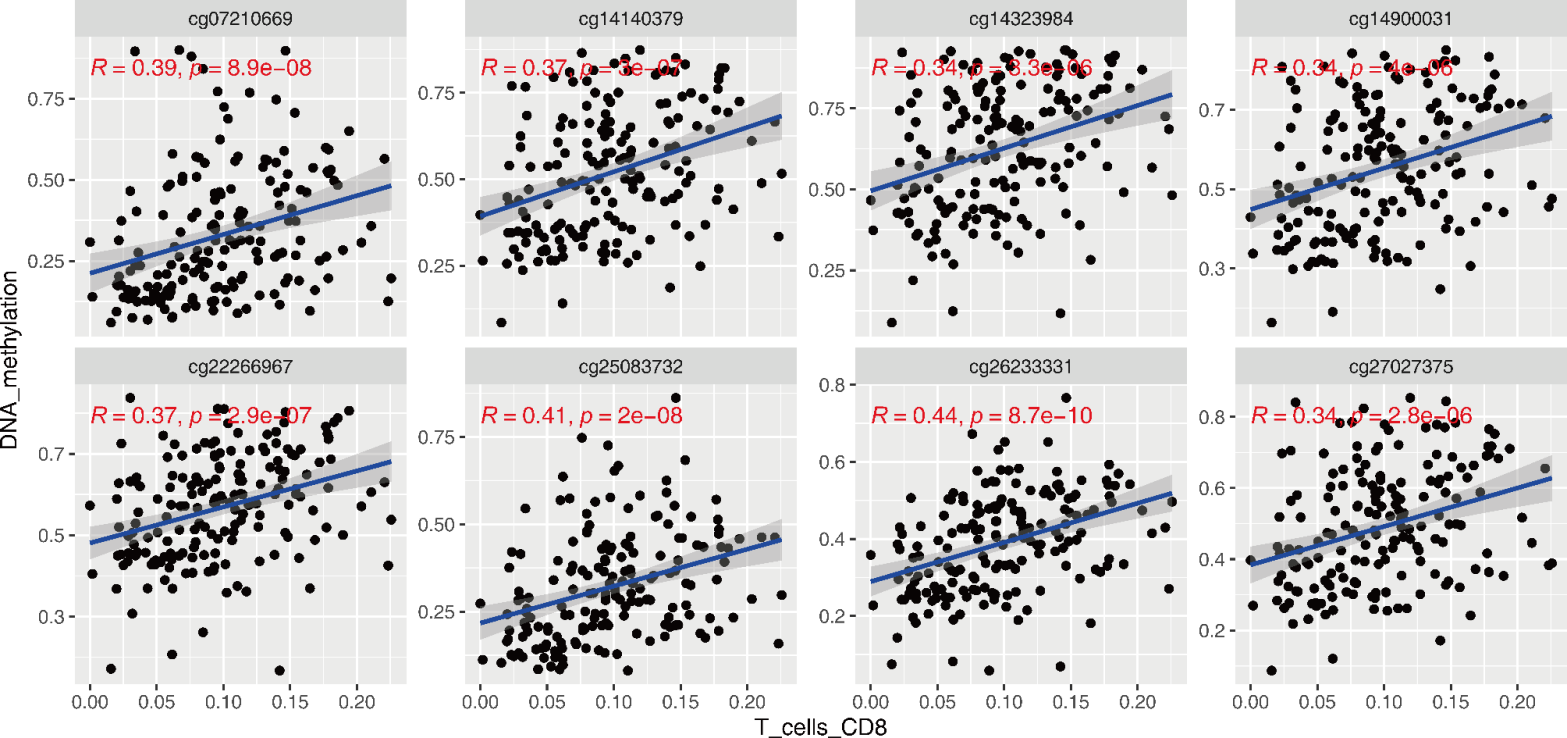
The correlation of CD8 + T cell infiltration and eight DNA methylation probs using *Spearman* analysis

The expression of mRNA for the gene *S100P* was found to have a negative correlation with the methylation levels of eight different probes: cg14323984, cg27027375, cg14900031, cg14140379, cg25083732, cg07210669, cg26233331, and cg22266967 (*P* < 0.001, r = -0.583; *P* < 0.001, r = -0.728; *P* < 0.001, r = -0.737; *P* < 0.001, r = -0.734; *P* < 0.001, r = -0.551; *P* < 0.001, r = -0.689; *P* < 0.001, r = -0.387; *P* < 0.001, r = -0.436; respectively; as shown in Fig. 7). Additionally, these eight probes were found to have an association with CD8 + T cell infiltration (Fig. 8).

### *S100P* overexpression at cellular and protein levels in pancreatic cancer

**Fig. 9:**
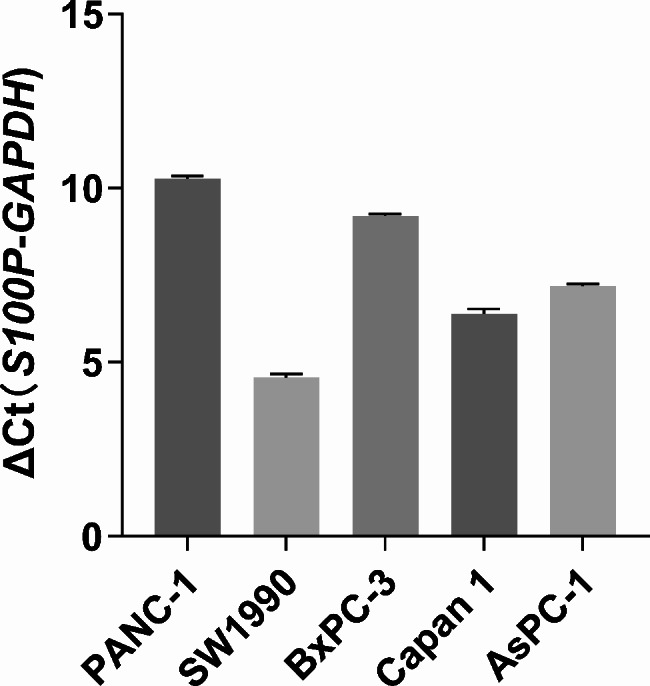
The expression of *S100P* in four pancreatic cancer cells using RT-PCR. ΔCt(*S100P*-*GAPDH*) ≦ 12 was regarded as high expression abundance

**Fig. 10:**
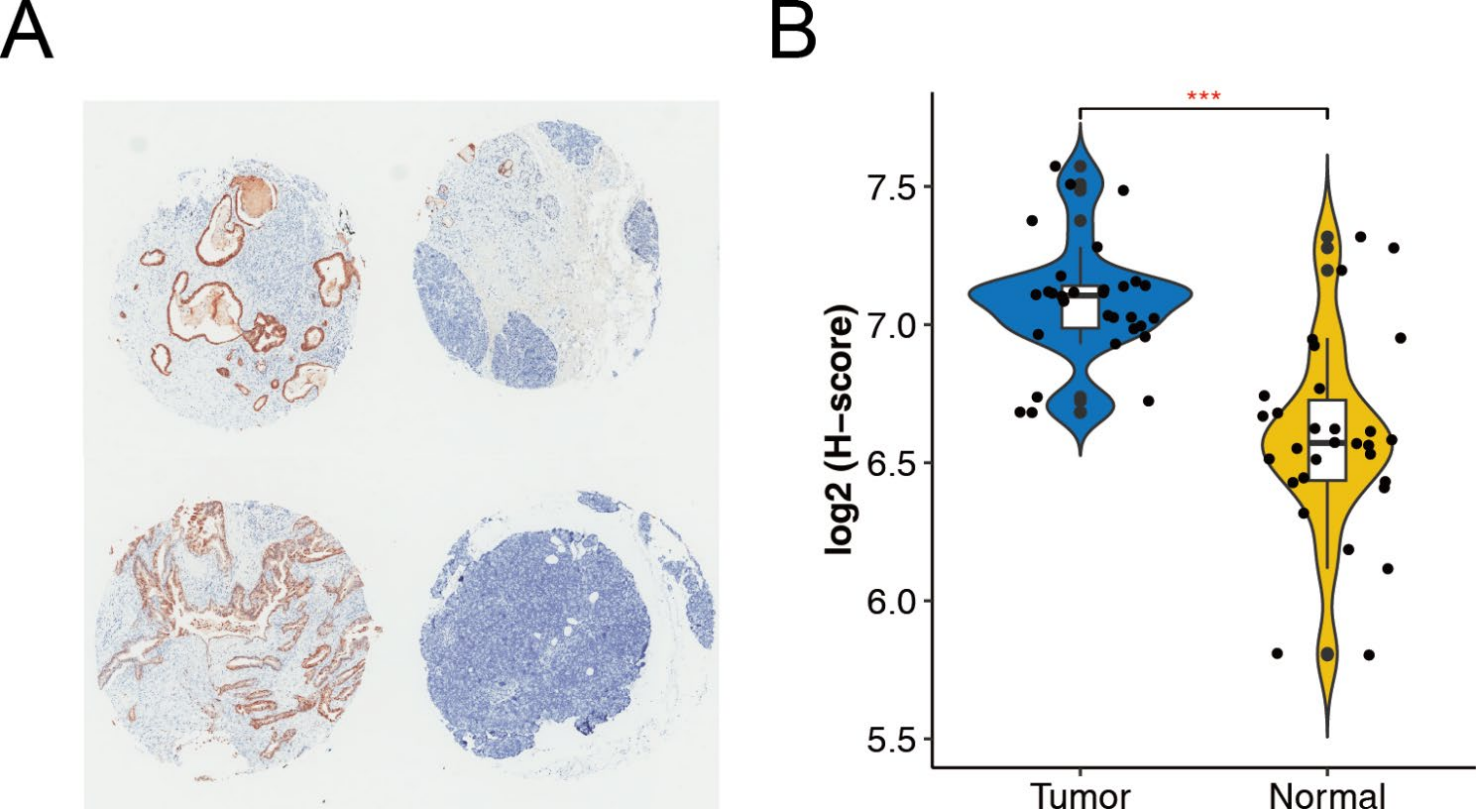
The expression of S100P in 30 pancreatic cancer tissues and 30 adjacent tissues using immunohistochemistry

After conducting experiments and analyzing data, it was found that *S100P* expression is elevated in pancreatic cancer at both cellular and protein levels. RT-PCR results indicated the upregulation of *S100P* in five pancreatic cancer cell lines (Fig. 9), and immunohistochemistry test images revealed higher expression of S100P in pancreatic cancer tissues than in adjacent tissues (Fig. 10). Additionally, the UALCAN dataset also demonstrated a significant increase in S100P expression in pancreatic cancer tissues, as compared to normal tissues (*P* < 0.05) (Figure S4).

## Discussion

Pancreatic cancer is widely regarded as one of the most malignant tumors, and the study of the TME is of paramount importance for uncovering the regulatory factors of pancreatic cancer therapy. Thus, this study aims to examine the possible role of *S100P* in the TME of pancreatic cancer in a systematic manner. Our findings demonstrate that *S100P* is highly expressed in pancreatic cancer and significantly correlates with CD8 + T cells. The expression of *S100P* is assessed at single-cell levels and validated in vitro RT-PCR and IHC experiments. Our findings indicated that *S100P* can potentially contribute to the immunosuppressive microenvironment associated with pancreatic cancer.

*S100P*, a 95-amino-acid member of the S100 protein family, has been previously established to promote pancreatic cancer growth, metastasis, and invasion [24]. Literature has shown that *S100P* is involved in the processes of angiogenesis, immune evasion, and can inactivate p53 [25, 26]. Recently, Zou et al. demonstrated that *S100P* was a novel immune-related biomarker and explored its prognostic significance [27]. Other members of the S100 family, such as *S100A2* [28], *S100A5* [29] and *S100A14* [30] has been confirmed to be involved in the regulation of immune microenvironment. In addition, Camara et al. proved that *S100P* inhibitors might provide a novel approach to treating pancreatic cancer [31]. However, the molecular mechanisms underlying the role of *S100P* in the TME remain poorly understood. We argue that the in-depth study of *S100P* can provide crucial insights and serve as the basis for the development of pancreatic cancer therapy.

Firstly, we comprehensively analyzed the *S100P* landscape in cancer, including its expression, survival, and clinical associations. Our results revealed diverse expression patterns of *S100P* across different cancer types, with high expression observed in pancreatic cancer and a correlation with poor prognosis. The credibility of these findings was strengthened through validation using multiple microarrays and CCLE datasets. Additionally, our analyses indicated that *S100P* expression is upregulated in patients with KRAS mutation and II-IV stage, suggesting its involvement in fundamental tumorigenic pathways in the pancreas. Enrichment analysis suggested a connection between *S100P* and immune-related signaling pathways, with *S100P* being listed in the ImmPort database as a relevant gene. Given the impact of tumor-infiltrating immune cells on cancer progression and therapy [32], further investigation into the role of *S100P* in the tumor microenvironment and its potential for immunotherapy is imperative.

Next, our study aimed to investigate the potential role of *S100P* in the immune microenvironment of pancreatic cancer. Notably, our results revealed a negative correlation between *S100P* expression levels and ImmuneScore, StromalScore, and ESTIMATEScore. Furthermore, we observed a significant association between increased *S100P* expression and reduced immune cell infiltration in two datasets. These findings suggest the involvement of *S100P* in immunosuppression-related pathways, indicating its potential relevance as an immunologic marker in pancreatic cancer. We further employed five different algorithms to quantify CD8 + T cell proportion, which supported our earlier findings of increased *S100P* expression being associated with lower CD8 + T cell infiltration. Using three scRNA-Seq datasets, we consistently observed a reduction in immune cell populations, including CD8 + T cells, CD4 + T cells, NK cells, B cells, and others, in patients with higher *S100P* expression. Previous studies have indicated that pancreatic cancer patients often exhibit low levels of tumor-infiltrating T cells, leading to poor response rates to immunotherapy [33]. Our analysis resonates with these findings, as we observed a high proportion of pancreatic cancer samples in the C3 subtype, consistent with reports that inflammation is likely to be involved in regulating the immunosuppressive microenvironment of pancreatic cancer [34]. Our data provide compelling evidence that *S100P* may play a crucial role in the remodeling of the TME and its association with immune “cold” tumors. Hence, targeting *S100P* may offer a promising strategy to convert the tumor immunologically “hot” and trigger an anticancer immune response.

The tumor immune microenvironment (TIME) has been demonstrated to be imperative for tumor development and clinical outcomes [35–37]. Thus, there is a possibility that therapeutic interventions targeting the TIME could serve as a promising immunotherapeutic approach for tumors [38]. To elucidate the potential of *S100P* as a pancreatic cancer immunotherapy, we analyzed the association between *S100P* expression and seven classic immune checkpoints in the TCGA-PAAD and E-MTAB-6134 cohorts. Our findings indicated that *S100P* was negatively linked with the expression of PD-1, *CTLA-4*, *IDO1*, and *LAG-3*. Moreover, the usefulness of TMB has already been established in predicting the response to cancer immunotherapy in numerous cancer types [39]. Our results showed that the correlation between *S100P* and TMB was 0.47, highlighting the potential of *S100P* as a complementary biomarker for predicting immunotherapy efficacy. Although further studies are necessary to validate these findings, emerging evidence portrays *S100P* as a probable player in the immune microenvironment and response to pancreatic cancer immunotherapy.

The present discussion aims to elucidate the relationship between *S100P* and the TME, which may be associated with Ca^2+^ signaling. Previous investigations have highlighted that Ca^2+^ can trigger crucial pathways for tumorigenesis, such as cellular motility, proliferation, and apoptosis [40]. Correspondingly, S100P, a Ca^2+^ binding protein, has been implicated in TME remodeling [41]. Moreover, Ca^2+^ signaling has regulatory effects on T cells as identified by Schlunck et al., who discovered an inverse correlation between Ca^2+^ mobilization and cell proliferation in T cells [42]. Hence, we postulate that *S100P* may impede immune cell infiltration in the peritumoral compartment by exerting influence on Ca^2+^ signaling. Recent research has demonstrated that Ca^2+^ signaling may display anti-tumor efficacy via modulation of the immune microenvironment [43, 44]. For instance, Rong et al. discovered that MGP promotes CD8 + T cell exhaustion in colorectal cancer by enriching intracellular free Ca^2+^ levels [45]. Furthermore, the mechanism of action of *S100P* may involve its interaction with the receptor for advanced glycation end products (*RAGE*) [46]. The interaction between *S100P* and *RAGE* can activate downstream signaling pathways, including Ca^2+^ signaling [47]. Additionally, *RAGE* has been reported to modulate the immune microenvironment by influencing the activation and function of immune cells, including T cells [48, 49]. Various studies have shown that inhibitors targeting *S100P* can improve the effectiveness of pancreatic cancer therapy [46, 50, 51]. Thus, regulating *S100P* expression through inhibitors could facilitate TME remodeling and provide potential therapeutic interventions for pancreatic cancer.

The above analysis provided evidence to support the hypothesis that higher *S100P* expression is linked to immune resistance in pancreatic cancer patients. Therefore, it is significant to investigate the underlying reasons for this expression pattern. Our findings indicated no mutations in the *S100P* gene in pancreatic cancer patients, but its expression was closely connected to methylation status. These results were consistent with previous research, suggesting that epigenetic mechanisms regulate *S100P* expression [52]. DNA methylation plays a critical role in the progression of cancer by inducing “exhaustion” in cytotoxic T cells, and may be used in combination with current immunotherapies [53, 54]. Moreover, we identified eight DNA methylation probes of *S100P* potentially associated with the TME in pancreatic cancer, as these methylation sites were positively correlated with CD8 + T cells. A recent study found that DNA methylation can be a predictive biomarker for response to immune checkpoint inhibitors [55]. Thus, downregulating the expression of *S100P* may enhance T cell immune activity and achieve anti-tumor immunotherapy objectives.

As far as we acknowledge, this study was the first comprehensive analysis to investigate the potential role of *S100P* in the immune microenvironment of pancreatic cancer. By incorporating various methodologies and in vitro validation, we obtained a multi-dimensional understanding of the involvement of *S100P*. However, some limitations of this study also warrant recognition. The relationship between *S100P* and the tumor environment is mainly based on observed correlations, and further mechanistic studies are needed to establish causation and fully elucidate the role of *S100P* in restructuring the tumor microenvironment in pancreatic cancer. Despite the lack of experimental validation and possible limitations, our study serves as a foundation for future investigations.

## Conclusion

Our study suggested a potential association between *S100P* and the restructuring of the tumor microenvironment in pancreatic cancer. The upregulation of *S100P* has been implicated in the immune dysfunction of pancreatic cancer, particularly in CD8 + T cells. Additionally, there is a potential for *S100P* to impact the regulation of pancreatic cancer through DNA hypomethylation. Further exploration of the molecular mechanisms underlying these findings should be conducted in future studies.

### Electronic supplementary material

Below is the link to the electronic supplementary material.

Supplementary Material 1

## Data Availability

All data generated or analyzed in this study are available upon reasonable request from the corresponding author. The relevant web links for accessing datasets are as follows: TCGA (https://portal.gdc.cancer.gov), GEO (https://www.ncbi.nlm.nih.gov/gds/), ArrayExpress (https://www.ebi.ac.uk/arrayexpress/), CCLE (https://sites.broadinstitute.org/ccle), UCSC Xena (https://xenabrowser.net/datapages/), UALCAN platform (http://ualcan.path.uab.edu/index.html), TISCH2 (http://tisch.comp-genomics.org/), MEXPRESS (https://www.mexpress.be/).

## Abbreviations

TME: tumor microenvironment
ssGSEA: single-sample gene set enrichment analysis
RT-PCR: Reverse transcription PCR
IHC: immunohistochemical
TMB: tumor mutation burden
IRGs: immunotherapy-related genes
TIME: tumor immune microenvironment
