# Supplementary material for: S100P as a potential biomarker for immunosuppressive microenvironment in pancreatic cancer: a bioinformatics analysis and in vitro study
Source: BMC Cancer. 2023 Oct 18;23:997. doi: 10.1186/s12885-023-11490-1 (PMC10585823; doi:10.1186/s12885-023-11490-1)
Supplement: Supplementary file 1 — Supplementary Material 1 [file 12885_2023_11490_MOESM1_ESM.docx]

# Supplementary materials

**
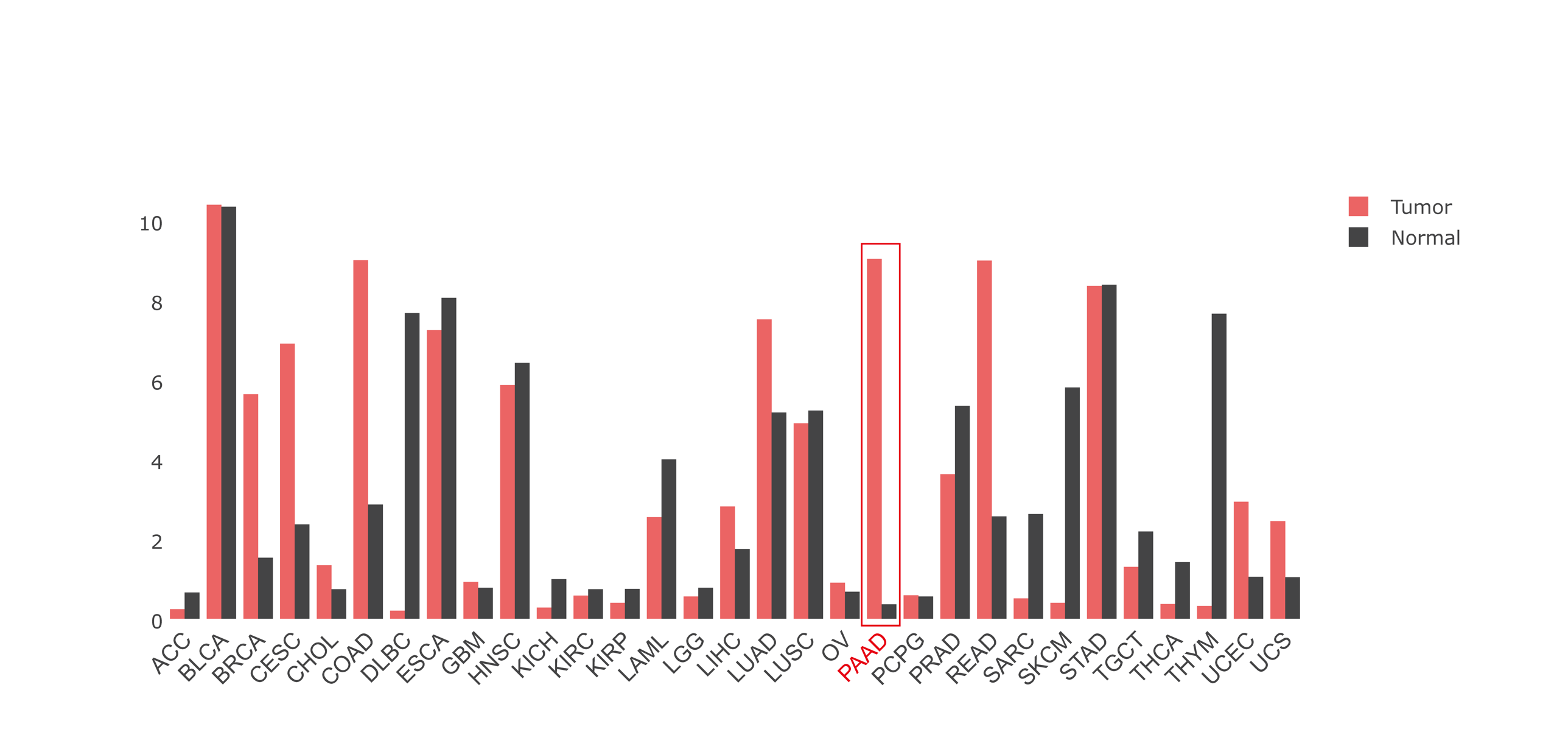
**

**Figure S1** The mRNA expression of *S100P* between tumor and adjacent tissues was assessed from TCGA and GTEx databases.





**Figure S2**  *S100P* expression in different clinicopathological stages of pancreatic cancer samples from the TCGA-PAAD.

**
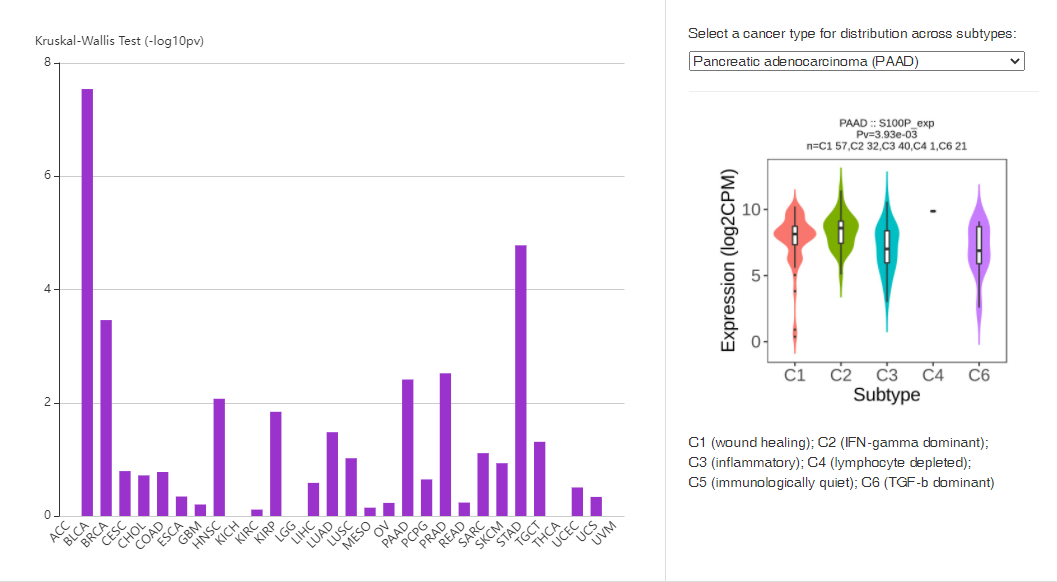
**

**Figure S3** Analysis of immune subtypes was assessed using the E-MTAB-6134 dataset.


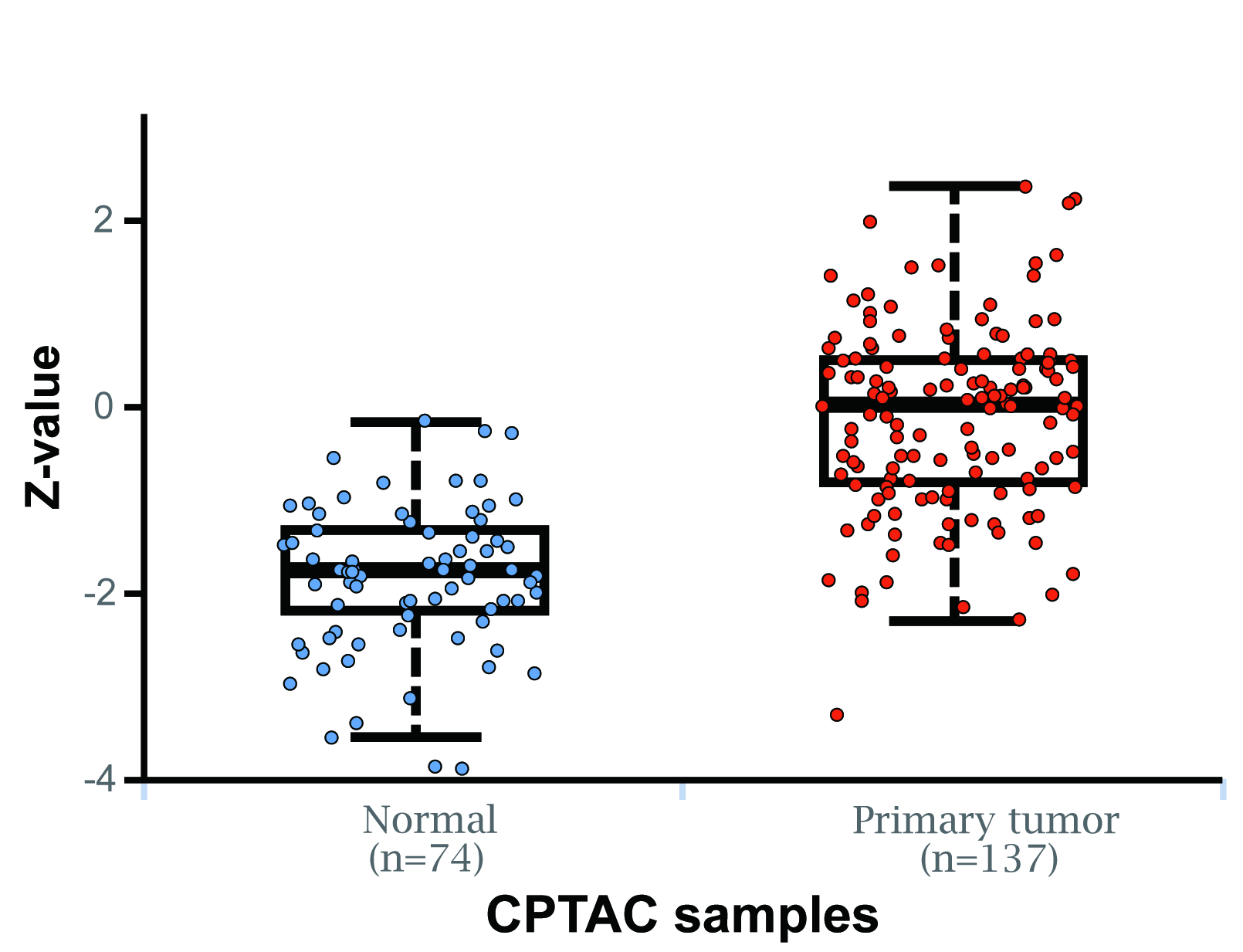


**Figure S4**  The protein expression of S100P in 137 primary pancreatic cancers and 74 adjacent tissues using the UALCAN dataset.
